# Supplementary material for: Towards unified AI-driven fracture mechanics: the extended deep energy method (XDEM)
Source: Nat Commun. 2026 Aug 15;17:8492. doi: 10.1038/s41467-026-76748-1 (PMC13484471; doi:10.1038/s41467-026-76748-1)
Supplement: Supplementary file 3 [file 41467_2026_76748_MOESM3_ESM.zip › pic/Results/Stress_intensity_factor/Cross/Figure_3.pdf]

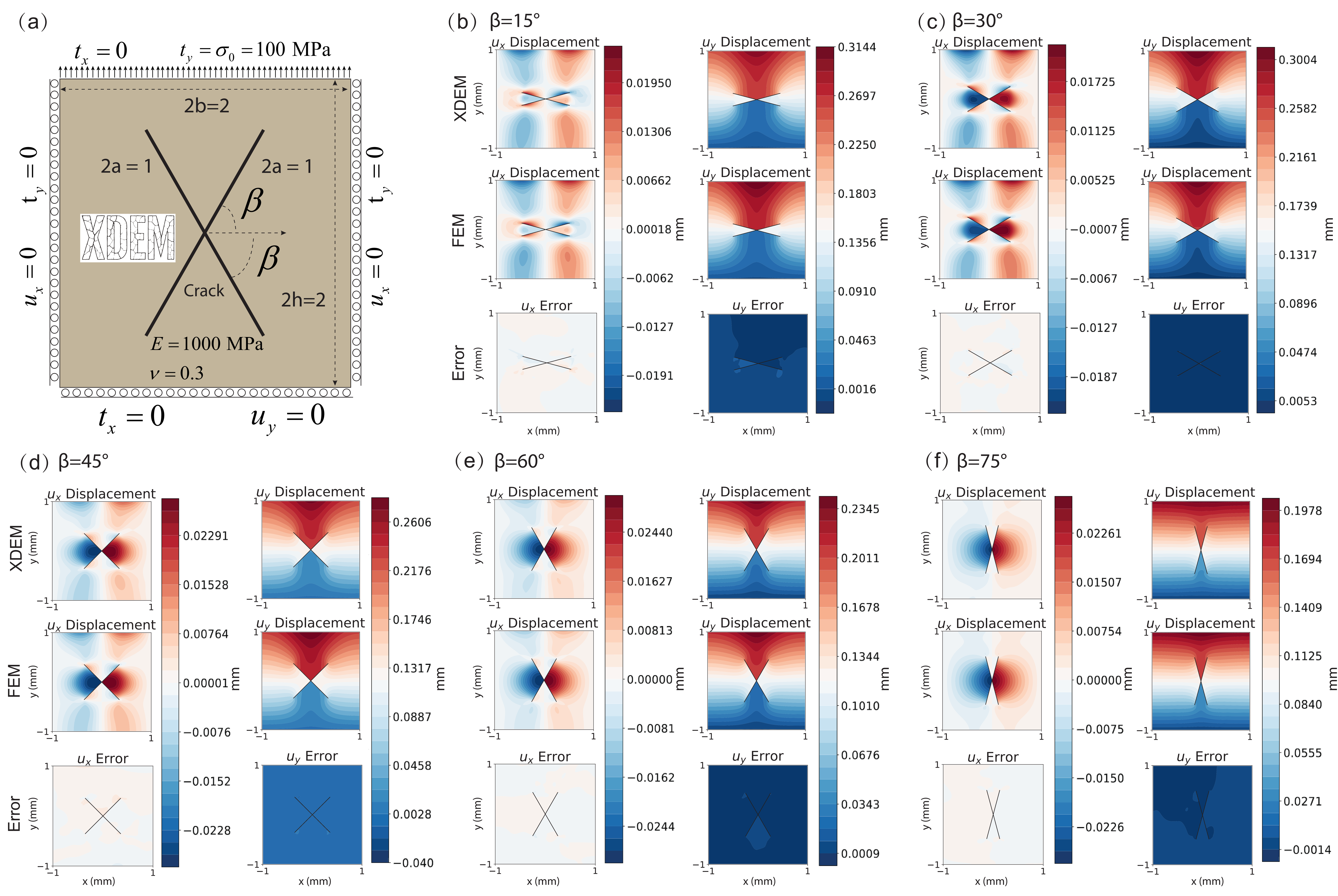
(a)
$t_x = 0$
$t_y = \sigma_0 = 100$ MPa
2b=2
2a = 1
2a = 1
β
β
XDEM
Crack
2h=2
$E = 1000$ MPa
$\nu = 0.3$
$t_y = 0$
$u_x = 0$
$t_y = 0$
$u_x = 0$
$t_x = 0$
$u_y = 0$
(b) β=15°
(c) β=30°
(d) β=45°
(e) β=60°
(f) β=75°
XDEM
FEM
Error
u_x Displacement
u_y Displacement
u_x Error
u_y Error
x (mm)
y (mm)
mm
0.01950
0.01306
0.00662
0.00018
−0.0062
−0.0127
−0.0191
0.3144
0.2697
0.2250
0.1803
0.1356
0.0910
0.0463
0.0016
0.01725
0.01125
0.00525
−0.0007
−0.0067
−0.0127
−0.0187
0.3004
0.2582
0.2161
0.1739
0.1317
0.0896
0.0474
0.0053
0.02291
0.01528
0.00764
0.00001
−0.0076
−0.0152
−0.0228
0.2606
0.2176
0.1746
0.1317
0.0887
0.0458
0.0028
−0.040
0.02440
0.01627
0.00813
0.00000
−0.0081
−0.0162
−0.0244
0.2345
0.2011
0.1678
0.1344
0.1010
0.0676
0.0343
0.0009
0.02261
0.01507
0.00754
0.00000
−0.0075
−0.0150
−0.0226
0.1978
0.1694
0.1409
0.1125
0.0840
0.0555
0.0271
−0.0014
